# Supplementary material for: The severity of microstrokes depends on local vascular topology and baseline perfusion
Source: eLife. 2021 May 18;10:e60208. doi: 10.7554/eLife.60208 (PMC8421069; doi:10.7554/eLife.60208)
Supplement: Supplementary file 1. — (a) Overview of the eight selection criteria used to analyze the impact of structural and functional characteristics on the severity of a microstroke. The different microstroke capillary (MSC) types are depicted in Figure 1a–d. For cases 1–7, the cortical depth selection criterion requires that only the source of the MSC be within the given range. For cases 8–12, at least one of the vertices should be within the given range, while the second one may be ±50 µm outside the given range. The mean and standard deviation (std) are calculated from the results of the baseline simulation for the eight chosen MSC per case. For the mean and std of the cortical depth the values of the source and the target vertex are both considered. The definition of the main branch is provided in the methods. DA: descending arteriole. AV: ascending venule. n: simulated number of MSCs per case. (b) Distribution of microstroke capillary (MSC) types over cortical depth for microvascular network (MVN) 1 and 2. AL: analysis layer. Abbreviations of the four MSC types: 2–2: 2-in-2-out, 2–1: 2-in-1-out, 1–2: 1-in-2-out, 1–1: 1-in-1-out. (c) Statistical results for the effect of the MSC type on the changes observed at different generations (Figure 1). The effect of the MSC type has been analyzed separately for the generations upstream (−5 to −1) and downstream (1 to 5) of the MSC. The statistical test has been performed in R with the function anova_test() as a two-way mixed ANOVA with Bonferroni correction. Upper: There is a significant simple main effect of the factor MSC type at all generations except generation 4 and 5. Lower table: Pairwise t-test to determine for which MSC types there is a significant difference in the changes observed per generation. Only pairs with a significant difference are listed. Case 1: 2-in-2-out, Case 2: 2-in-1-out, Case 3: 1-in-2-out, Case 4: 1-in-1-out. p-adj.: adjusted p-value, sign: significance. (d) Statistical results for the effect of the MSC type on the changes [file elife-60208-supp1.docx]

**Supplementary File - The severity of microstrokes depends on local vascular topology and baseline perfusion.**

Franca Schmid^1,2^, Giulia Conti^2^, Patrick Jenny^2^, Bruno Weber^1^

^1^ Institute of Pharmacology and Toxicology, University of Zurich, Winterthurerstrasse 190, CH-8057 Zurich, Switzerland

^2^ Institute of Fluid Dynamics, ETH Zurich, Sonneggstrasse 3, CH-8092 Zurich, Switzerland

Supplementary File 1a Overview of the eight selection criteria used to analyse the impact of structural and functional characteristics on the severity of a microstroke. The different microstroke capillary (MSC) types are depicted in Figure 1a-d. For cases 1-7 the cortical depth selection criterion requires that only the source of the MSC be within the given range. For cases 8-12 at least one of the vertices should be within the given range, while the second one may be ±50 µm outside the given range. The mean and standard deviation (std) are calculated from the results of the baseline simulation for the eight chosen MSC per case. For the mean and std of the cortical depth the values of the source and the target vertex are both considered. The definition of the main branch is provided in the methods. DA: descending arteriole. AV: ascending venule. n: simulated number of MSCs per case.

|  |  | Flow rate [µm^3^ms^-1^] | | | Cortical depth [µm] | | |  |  |
| --- | --- | --- | --- | --- | --- | --- | --- | --- | --- |
| Case | MSC type | min | max | mean ± std | min | max | mean ± std | Vessels to main branch of DA/AV | n |
| 1 | *2-in-2-out* | 0.1 | 4.0 | 2.58 ± 0.82 | 400 | 700 | 564 ± 73 | > 3 (DA), >3 (AV) | 27 |
| 2 | *2-in-1-out* | 0.1 | 4.0 | 2.45 ± 0.92 | 400 | 700 | 563 ± 85 | > 3 (DA), >3 (AV) | 20 |
| 3 | *1-in-2-out* | 0.1 | 4.0 | 2.32 ± 1.04 | 400 | 700 | 563 ± 77 | > 3 (DA), >3 (AV) | 22 |
| 4 | *1-in-1-out* | 0.1 | 4.0 | 1.72 ± 0.74 | 400 | 700 | 552 ± 80 | > 3 (DA), >3 (AV) | 20 |
|  |  |  |  |  |  |  |  |  |  |
| 5 | *2-in-2-out* | 6.6 | 25 | 9.84 ± 3.70 | 400 | 700 | 533 ± 75 | > 3 (DA), >3 (AV) | 20 |
|  |  |  |  |  |  |  |  |  |  |
| 6 | *2-in-2-out* | 0.1 | 7.0 | 3.67 ± 1.20 | 200 | 800 | 603 ± 155 | 2-3 (DA), >3 (AV) | 15 |
| 7 | *2-in-2-out* | 0.1 | 7.0 | 3.15 ± 1.47 | 200 | 800 | 462 ± 142 | > 7 (DA), >3 (AV) | 19 |
|  |  |  |  |  |  |  |  |  |  |
| 8 | *2-in-2-out* | 0.1 | 7.0 | 3.43 ± 1.67 | 0 | 200 | 93 ± 50 | > 3 (DA), >3 (AV) | 12 |
| 9 | *2-in-2-out* | 0.1 | 7.0 | 4.42 ± 1.77 | 200 | 400 | 306 ± 46 | > 3 (DA), >3 (AV) | 12 |
| 10 | *2-in-2-out* | 0.1 | 7.0 | 3.22 ± 1.53 | 400 | 600 | 511 ± 51 | > 3 (DA), >3 (AV) | 18 |
| 11 | *2-in-2-out* | 0.1 | 7.0 | 2.66 ± 0.66 | 600 | 800 | 667 ± 54 | > 3 (DA), >3 (AV) | 12 |
| 12 | *2-in-2-out* | 0.1 | 7.0 | 1.38 ± 0.98 | 800 | 100 | 900 ± 53 | > 3 (DA), >3 (AV) | 12 |

Supplementary File 1b Distribution of microstroke capillary (MSC) types over cortical depth for microvascular network (MVN) 1 and 2. AL: Analysis Layer. Abbreviations of the four MSC-types: 2-2: *2-in-2-out*, 2-1: *2-in-1-out*, 1-2: *1-in-2-out*, 1-1: *1-in-1-out*.

| MVN | AL | Cortical depth [µm] | | Total number of MSC | Frequency of different MSC types [%] | | | |
| --- | --- | --- | --- | --- | --- | --- | --- | --- |
|  |  | min | max |  | *2-2* | *2-1* | *1-2* | *1-1* |
| 1 | 1 | 0 | 200 | 832 | 8.1 | 20.4 | 23.3 | 45.9 |
| 1 | 2 | 200 | 400 | 1004 | 8.1 | 15.3 | 31.1 | 43.7 |
| 1 | 3 | 400 | 600 | 1137 | 10.2 | 18.5 | 25.6 | 44.2 |
| 1 | 4 | 600 | 800 | 916 | 11.8 | 20.7 | 23.5 | 42.8 |
| 1 | 5 | 800 | 1000 | 802 | 12.1 | 20.0 | 27.2 | 39.8 |
|  |  |  |  |  |  |  |  |  |
| 2 | 1 | 0 | 200 | 1541 | 4.9 | 19.5 | 30.3 | 42.7 |
| 2 | 2 | 200 | 400 | 1913 | 4.3 | 17.5 | 34.3 | 41.4 |
| 2 | 3 | 400 | 600 | 2046 | 6.1 | 18.2 | 30.7 | 42.7 |
| 2 | 4 | 600 | 800 | 1816 | 7.0 | 19.3 | 28.0 | 43.2 |
| 2 | 5 | 800 | 1000 | 1314 | 8.7 | 17.1 | 29.6 | 43.2 |

Supplementary File 1c Statistical results for the effect of the MSC-type on the changes observed at different generations (Figure 1). The effect of the MSC-type has been analysed separately for the generations up- (-5 to -1) and downstream (1 to 5) of the MSC. The statistical test has been performed in R with the function anova_test() as a two-way mixed ANOVA with Bonferroni correction. Upper table: There is a significant simple main effect of the factor MSC-type at all generations except generation 4 and 5. Lower table: Pairwise t-test to determine for which MSC-types there is a significant difference in the changes observed per generation. Only pairs with a significant difference are listed. Case 1: *2-in-2-out*, Case 2: *2-in-1-out*, Case 3: *1-in-2-out*, Case4*: 1-in-1-out*. p-adj.: adjusted p-value, sign: significance.

| Generation | -5 | -4 | -3 | -2 | -1 | 1 | 2 | 3 | 4 | 5 |
| --- | --- | --- | --- | --- | --- | --- | --- | --- | --- | --- |
| p-adj. | 4.0e^-2^ | 4.4e^-5^ | 4.3e^-4^ | 2.3e^-5^ | 3.6e^-9^ | 5.0e^-3^ | 2.5e^-3^ | 1.5e^-2^ | 1.1e^-1^ | 8.0e^-2^ |
| sign. | * | **** | *** | **** | **** | ** | ** | * | ns | ns |

| Generation | Pair - Cases | p-adj. | sign. | Generation | Pair – Cases | p-adj. | sign. |
| --- | --- | --- | --- | --- | --- | --- | --- |
| -5 | 1 vs 4 | 2.1e^-2^ | * | 5 | 1 vs 4 | 1.4e^-2^ | * |
|  | 2 vs 4 | 4.3e^-2^ | * |  |  |  |  |
| -4 | 1 vs 3 | 8.4e^-3^ | ** | 4 | 1 vs 4 | 1.9e^-2^ | * |
|  | 2 vs 3 | 2.0e^-4^ | *** |  |  |  |  |
|  | 1 vs 4 | 8.2e^-3^ | ** |  |  |  |  |
|  | 2 vs 4 | 2.1e^-4^ | *** |  |  |  |  |
| -3 | 1 vs 3 | 1.6e^-2^ | * | 3 | 2 vs 4 | 2.5e^-3^ | ** |
|  | 2 vs 3 | 8.1e^-4^ | *** |  |  |  |  |
|  | 1 vs 4 | 3.2e^-2^ | * |  |  |  |  |
|  | 2 vs 4 | 1.8e^-3^ | ** |  |  |  |  |
| -2 | 1 vs 3 | 1.4e^-4^ | *** | 2 | 1 vs 4 | 9.4e^-4^ | *** |
|  | 2 vs 3 | 8.9e^-3^ | *** |  | 2 vs 4 | 1.8e^-4^ | ** |
|  | 1 vs 4 | 9.7e^-4^ | *** |  |  |  |  |
|  | 2 vs 4 | 4.4e^-3^ | ** |  |  |  |  |
| -1 | 1 vs 3 | 2.9e^-7^ | **** | 1 | 1 vs 4 | 6.7e^-4^ | *** |
|  | 2 vs 3 | 1.7e^-5^ | **** |  | 2 vs 4 | 2.9e^-2^ | * |
|  | 1 vs 4 | 8.5e^-7^ | **** |  |  |  |  |
|  | 2 vs 4 | 3.7e^-5^ | **** |  |  |  |  |

Supplementary File 1d Statistical results for the effect of the MSC-type on the changes in inflow rate for analysis boxes of different volumes (Figure 2b-e). The statistical test has been performed in R with the function anova_test() as a two-way mixed ANOVA with Bonferroni correction. Upper table: There is a significant simple main effect of the factor MSC-type for all volume factors <2.75. Lower table: Pairwise t-test to determine for which MSC-types there is a significant difference in the changes observed per volume factor. Only pairs with a significant difference are listed. Case 1: *2-in-2-out*, Case 2: *2-in-1-out*, Case 3: *1-in-2-out*, Case4*: 1-in-1-out*. p-adj.: adjusted p-value, sign: significance.

| Volume factor | 1.0 | 1.25 | 1.5 | 1.75 | 2.0 | 2.25 | 2.5 | 2.75 | 3.0 |
| --- | --- | --- | --- | --- | --- | --- | --- | --- | --- |
| p-adj. | 5.5e^-4^ | 1.4e^-3^ | 4.6e^-3^ | 2.7e^-2^ | 1.8e^-2^ | 2.7e^-2^ | 4.5e^-2^ | 9.0e^-2^ | 9.9e^-2^ |
| sign. | *** | ** | ** | * | * | * | * | ns | ns |

| Volume factor | Pair - Cases | p-adj. | sign. | Volume factor | Pair – Cases | p-adj. | sign. |
| --- | --- | --- | --- | --- | --- | --- | --- |
| 1.0 | 1 vs 3 | 3.5e^-3^ | ** | 1.75 | 1 vs 4 | 8.4e^-3^ | ** |
|  | 1 vs 4 | 1.3e^-4^ | *** | 2.0 | 1 vs 3 | 4.2e^-2^ | * |
|  | 2 vs 4 | 2.2e^-2^ | * |  | 1 vs 4 | 4.3e^-3^ | ** |
| 1.25 | 1 vs 3 | 1.1e^-2^ | * |  | 2 vs 4 | 4.3e^-2^ | * |
|  | 1 vs 4 | 2.1e^-4^ | *** | 2.25 | 1 vs 4 | 1.3e^-2^ | * |
|  | 2 vs 4 | 2.8e^-2^ | * | 2.5 | 1 vs 4 | 1.4e^-2^ | * |
| 1.5 | 1 vs 3 | 4.2e^-2^ | * |  | 2 vs 4 | 4.9e^-2^ | * |
|  | 1 vs 4 | 4.2e^-4^ | *** |  |  |  |  |
|  | 2 vs 4 | 4.3e^-2^ | * |  |  |  |  |

Supplementary File 1e Statistical results for the characteristics of different MSC-types (Figure 5f-k). The statistical test has been performed in with the Python library scipy.stats. The Kruskal-Wallis test showed a significant difference between supplied tissue volume, flow rate and number of paths in both microvascular networks (MVNs, all p-values <0.001). Below the p-values of the pairwise comparison with the Mann-Whitney U test are listed. Upper table: p-values for MVN1. Lower table: p-values for MVN2. Abbreviations for the MSC-types: *2-2:* *2-in-2-out*, *2-1: 2-in-1-out*, *1-2: 1-in-2-out*, *1-1:* *1-in-1-out*. ns: not significant.

| **MVN1** | Supplied tissue volume | | | Flow rate | | | Number of paths | | |
| --- | --- | --- | --- | --- | --- | --- | --- | --- | --- |
|  | *2-2* | *2-1* | *1-2* | *2-2* | *2-1* | *1-2* | *2-2* | *2-1* | *1-2* |
| *2-1* | 0.03 | - | - | <1e^-3^ | - | - | <1e^-3^ | - | - |
| *1-2* | 0.04 | ns | - | ns | <1e^-3^ | - | <1e^-3^ | <1e^-3^ | - |
| *1-1* | <1e^-3^ | <1e^-3^ | <1e^-3^ | <1e^-3^ | <1e^-3^ | <1e^-3^ | <1e^-3^ | <1e^-3^ | <1e^-3^ |

| **MVN2** | Supplied tissue volume | | | Flow rate | | | Number of paths | | |
| --- | --- | --- | --- | --- | --- | --- | --- | --- | --- |
|  | *2-2* | *2-1* | *1-2* | *2-2* | *2-1* | *1-2* | *2-2* | *2-1* | *1-2* |
| *2-1* | 0.03 | - | - | ns | - | - | <1e^-3^ | - | - |
| *1-2* | <1e^-3^ | <1e^-3^ | - | <1e^-3^ | <1e^-3^ | - | <1e^-3^ | ns | - |
| *1-1* | <1e^-3^ | <1e^-3^ | <1e^-3^ | <1e^-3^ | <1e^-3^ | <1e^-3^ | <1e^-3^ | <1e^-3^ | <1e^-3^ |
|  |  |  |  |  |  |  |  |  |  |

Supplementary File 1f Absolute differences between averaged flow rates in all capillaries at two time points t1 and t2. The time difference between the two time points is 20s. In the left panel the absolute differences for an averaging interval of 10 turnover times (ToT) are displayed. In the middle and the left panel the differences for averaging intervals of 5 ToTs and 3 ToTs are shown. The absolute differences between the averaged results increase for smaller averaging intervals. For an averaging interval of 10 ToT for 94% of all vessels the absolute difference is smaller than 0.1 µm^3^ms^-1^. This value decreases to 91% and 87% for an averaging interval of 5 ToT and 3 ToT, respectively.
