# Supplementary material for: The severity of microstrokes depends on local vascular topology and baseline perfusion
Source: eLife. 2021 May 18;10:e60208. doi: 10.7554/eLife.60208 (PMC8421069; doi:10.7554/eLife.60208)
Supplement: Transparent reporting form [file elife-60208-transrepform.pdf]

Westbrook Centre
Milton Road
Cambridge CB4 1YG, UK
P 01223 855340
W elifesciences.org
T @elife

eLife's transparent reporting form

We encourage authors to provide detailed information *within their submission* to facilitate the interpretation and replication of experiments. Authors can upload supporting documentation to indicate the use of appropriate reporting guidelines for health-related research (see EQUATOR Network), life science research (see the BioSharing Information Resource), or the ARRIVE guidelines for reporting work involving animal research. Where applicable, authors should refer to any relevant reporting standards documents in this form.

If you have any questions, please consult our Journal Policies and/or contact us: editorial@elifesciences.org.

Sample-size estimation

- You should state whether an appropriate sample size was computed when the study was being designed
- You should state the statistical method of sample size computation and any required assumptions
- If no explicit power analysis was used, you should describe how you decided what sample (replicate) size (number) to use

Please outline where this information can be found within the submission (e.g., sections or figure legends), or explain why this information doesn't apply to your submission:

No power analysis was used to estimate the sample size.
To further comment on the sample size it should be distinguished between analyses focusing on 1) the entire capillary bed and 2) the comparison of microstroke cases.

- 1) Investigations focusing on the entire capillary bed:
  The microvascular networks contain several thousand capillaries, i.e. the sample size is very large and allows conclusion on general characteristics of the capillary bed. The precise sample size is given in the figure legends.
- 2) Comparison of microstroke cases:
  We chose >= 12 different microstroke capillaries as sample size for one microstroke case and >=20 microstroke capillaries for the most relevant cases. Here, the sample size is a compromise between total computational cost and observable differences between cases with different topological configurations or further factors influencing the severity of a microstroke. It is important to note that even if the sample size per microstroke case is >=12 the total number of capillaries analyzed per case is significantly larger. This is because we also consider capillaries up to five generations apart from the microstroke capillary and within an analysis box of > 200,000 μm^3.

Replicates

- You should report how often each experiment was performed
- You should include a definition of biological versus technical replication
- The data obtained should be provided and sufficient information should be provided to indicate the number of independent biological and/or technical replicates
- If you encountered any outliers, you should describe how these were handled
- Criteria for exclusion/inclusion of data should be clearly stated
- High-throughput sequence data should be uploaded before submission, with a private link

eLife Sciences Publications, Ltd is a limited liability non-profit non-stock corporation incorporated in the State of Delaware, USA, with company number 5030732, and is registered in the UK with company number FC030576 and branch number BR015634 at the address Westbrook Centre, Milton Road, Cambridge CB4 1YG.

for reviewers provided (these are available from both GEO and ArrayExpress)

Please outline where this information can be found within the submission (e.g., sections or figure legends), or explain why this information doesn't apply to your submission:

- The need to perform replicates does not apply for our study. The time-averaged simulation results are deterministic such that replicates are not necessary.
- The selection criteria for capillaries considered in the study is described in 5. Methods → 5.2 Microstroke simulations. Additional information is provided in Supplementary File 1a and the Figure legends.
- Outliers are not excluded for the computation of the median or the boxplots. However, for illustrative purposes some outliers might not be displayed. This is clearly stated in the Figure legend (e.g. Fig 4c and Figure 6 – supplement figure 1). Outliers are mostly capillaries with large relative changes due to low baseline values.

Statistical reporting

- Statistical analysis methods should be described and justified
- Raw data should be presented in figures whenever informative to do so (typically when N per group is less than 10)
- For each experiment, you should identify the statistical tests used, exact values of N, definitions of center, methods of multiple test correction, and dispersion and precision measures (e.g., mean, median, SD, SEM, confidence intervals; and, for the major substantive results, a measure of effect size (e.g., Pearson's r, Cohen's d)
- Report exact p-values wherever possible alongside the summary statistics and 95% confidence intervals. These should be reported for all key questions and not only when the p-value is less than 0.05.

Please outline where this information can be found within the submission (e.g., sections or figure legends), or explain why this information doesn't apply to your submission:

- Within the figures we provide the raw data for each microstroke simulation. Whenever possible, we also provide the raw data for individual capillaries.
- To conclude that changes in flow rate between the baseline and the stroke scenario are significant, we analyze the baseline fluctuations of the flow field and derive a robust criterion for the relative change in flow rate. The approach is described in 5.Methods →5.3 Thresholded relative change and Supplementary File 1f.
- The statistical tests are summarized in the figure legends. The summary includes N, the statistical test employed and the resulting p-value. More detailed results are presented in Supplementary File 1c-e. In the Methods – Section 5.11 Statistics we provide an overview of all statistical tests employed. Within the text we provide the p-values for the most relevant conclusions.

(For large datasets, or papers with a very large number of statistical tests, you may upload a single table file with tests, Ns, etc., with reference to sections in the manuscript.)

Group allocation

- Indicate how samples were allocated into experimental groups (in the case of clinical studies, please specify allocation to treatment method); if randomization was used, please also state if restricted randomization was applied
- Indicate if masking was used during group allocation, data collection and/or data analysis

eLife Sciences Publications, Ltd is a limited liability non-profit non-stock corporation incorporated in the State of Delaware, USA, with company number 5030732, and is registered in the UK with company number FC030576 and branch number [illegible]

Please outline where this information can be found within the submission (e.g., sections or figure legends), or explain why this information doesn't apply to your submission:

For analysis purposes individual capillaries were assigned to different group (e.g. based on baseline flow rate). The precise criteria are described in 5. Methods → 5.2 Microstroke simulations and summarized in Supplementary File 1a.

Additional data files ("source data")

- We encourage you to upload relevant additional data files, such as numerical data that are represented as a graph in a figure, or as a summary table
- Where provided, these should be in the most useful format, and they can be uploaded as "Source data" files linked to a main figure or table
- Include model definition files including the full list of parameters used
- Include code used for data analysis (e.g., R, MatLab)
- Avoid stating that data files are "available upon request"

Please indicate the figures or tables for which source data files have been provided:

We did not yet upload any source files. However, we would like to upload the time-averaged simulations results including analysis scripts upon acceptance of our manuscript.

eLife Sciences Publications, Ltd is a limited liability non-profit non-stock corporation incorporated in the State of Delaware, USA, with company number 5030732, and is registered in the UK with company number FC030576 and branch number [illegible]
